# Supplementary material for: Ten years of visual field change in people living with diabetes: A prospective longitudinal study
Source: PLoS One. 2025 Mar 25;20(3):e0320285. doi: 10.1371/journal.pone.0320285 (PMC11936255; doi:10.1371/journal.pone.0320285)
Supplement: Attachment — Submitted filename: Response to Reviewers.docx [file pone.0320285.s001.docx]

We thank the editor and the reviewers for the comments. The thorough critical reviews are highly appreciated. Please, se our replies below.

Yours sincerely,

Karl-Johan Hellgren, MD, PhD

## Comments and replies to Academic Editor Daisuke Nagasato

1. Please ensure that your manuscript meets PLOS ONE's style requirements, including those for file naming. The PLOS ONE style templates can be found at

<https://journals.plos.org/plosone/s/file?id=wjVg/PLOSOne_formatting_sample_main_body.pdf> and

<https://journals.plos.org/plosone/s/file?id=ba62/PLOSOne_formatting_sample_title_authors_affiliations.pdf>

**Reply:** We have ensured that the manuscript meets the requirements.

-------------------

2. Thank you for stating the following in the Competing Interests section:

"I have read the journal's policy and the authors of this manuscript have the following competing interests: Hellgren KJ has no competing interests. Bengtsson B receives royalties from Carl Zeiss Meditec and has been a consultant of Carl Zeiss Meditec"

Please confirm that this does not alter your adherence to all PLOS ONE policies on sharing data and materials, by including the following statement: ""This does not alter our adherence to  PLOS ONE policies on sharing data and materials.” (as detailed online in our guide for authors http://journals.plos.org/plosone/s/competing-interests).  If there are restrictions on sharing of data and/or materials, please state these. Please note that we cannot proceed with consideration of your article until this information has been declared.

Please include your updated Competing Interests statement in your cover letter; we will change the online submission form on your behalf.

**Reply:** The Competing Interest statement does not alter our adherence PLOS ONE policies and the statement have changed as proposed and included in the cover letter:

“I have read the journal's policy and the authors of this manuscript have the following competing interests: Hellgren KJ has no competing interests. Bengtsson B receives royalties from Carl Zeiss Meditec and has been a consultant of Carl Zeiss Meditec. This does not alter our adherence to PLOS ONE policies on sharing data and materials.”

-------------------

3. In the online submission form, you indicated that "The data, which contains sensitive personal data, is not publicly available due to legal and ethical restrictions. Deidentified participant data is available for researchers after a reasonable request to the authors and the data owner, County Council of Värmland. Requests may be sent to the corresponding author, Karl-Johan Hellgren."

All PLOS journals now require all data underlying the findings described in their manuscript to be freely available to other researchers, either 1. In a public repository, 2. Within the manuscript itself, or 3. Uploaded as supplementary information.

This policy applies to all data except where public deposition would breach compliance with the protocol approved by your research ethics board. If your data cannot be made publicly available for ethical or legal reasons (e.g., public availability would compromise patient privacy), please explain your reasons on resubmission and your exemption request will be escalated for approval.

**Reply:** The data cannot be shared publicly because it contains extensive amount of sensitive personal data on a relatively small number of individuals from a specified and local area which also can be used to identify people, e.g. age, sex, diabetes type, diabetes duration, repeated biometric measurements and updates on diseases and medications. According to the ethical approvals from 2004, 2006 and 2015, data access is currently limited to participating researchers. Researchers may request deidentified data from the data owner, County Council of Värmland (Region Värmland) https://regionvarmland.se, datauttagforskning@regionvarmland.se or contact the corresponding author for guidance. Access may require an approved application from the Swedish Ethical Review Authority (https://etikprovningsmyndigheten.se).

-------------------

## Comments and replies to Reviewer #1

1. Line 108-109: The authors state about the definition of type 1 diabetes. However, the diagnosis of type 1 diabetes should be based on internal medicine criteria. It is not uncommon for individuals under 30 to be diagnosed with type 2 diabetes and subsequently require insulin therapy. If it is unclear whether patients have type 1 or type 2 diabetes, and if this distinction is not a crucial factor in the context of your study, it is advisable to state this uncertainty explicitly.

**Reply:** We do agree that our definition of diabetes type may imply some uncertainty in proportion of diabetes type in patient characteristics, but this uncertainty did not affect any other results since diabetes type was not included in any of the statistical models and thus not an outcome variable. A review of medical records to possibly confirm internal medicine criteria at diagnosis as type 1 or type 2 was not performed in this prospective study, since most patients were diagnosed with diabetes years before the start of the current study.

-------------------

2. The authors position the reliability of their analysis based on the finding that excluding the three eyes with the poorest final visual acuity and moderate or worse DR did not alter the results. However, is there a rationale for excluding these three eyes? Additionally, why were only cases with ETDRS levels of 43 or higher excluded? If there is previous literature or a basis for this decision, it should be stated.

**Reply:** No association was seen between visual field deterioration and DR change. Most DR change occurred between no to mild DR and vice versa, thus the result indicates a visual dysfunction to occur early in a microvascular perspective. Three eyes progressed to level 43, moderate DR, or worse. An additional analysis omitting these eyes were conducted to ensure that the results were not driven by these eyes. A clarification has been added to the Result section, subheading Visual fields, Revised Manuscript with Track Changes, line 254 – 255.

-------------------

3. In Figure 2, the authors describe the correlation between HbA1c levels and the number of visual field deterioration points across different durations of diabetes. At what point in time were these HbA1c values measured? Did you consider the fluctuations in HbA1c levels? If so, please discuss this in the manuscript.

**Reply:** The model included all HbA1c from each follow-up visit, i.e. 1, 1 ½, 2, 2 ½, 3, 4, 5, 9 and 10 years from baseline. All patient series of HbA1c values was included in the analysis. A clarification has been inserted in the Method section, subheading Statistics, Revised Manuscript with Track Changes, Line 212 - 214.

“Thus, the model included all values of the outcome variable, i.e. number of deteriorated test points, and all measures of the potential predictors from each follow-up visit, e.g. HbA1c values at 1, 1 ½, 2, 2 ½, 3, 4, 5, 9 and 10 years. “

-------------------

4. Line 259: It is stated that cataracts or cataract surgery affected the visual field test results in two cases. Were these two cases excluded from the analysis? If they were not, what is the reason?

**Reply:** No, the two cases were not excluded, but a rerun of the analysis excluding these cases revealed a similar result (deteriorated test points increased by IRR 1.118 (1.077 – 1.161) compared to 1.110 (1.069 – 1.153)). Neither did the result change in any meaningful way if all nine subjects which had cataract surgery during follow-up were excluded from the analysis (deteriorated test points increased by IRR 1.114 (1.068 – 1.162).

A clarification has been made and by rephrasing the sentence, Result section, Manuscript with Track Changes, line 303 – 304.

From “There was no effect of cataract or cataract surgery on the visual field test results in seven of the nine eyes.”

To “The results were similar when these cases were excluded.”

-------------------

5. The finding that there is a negative correlation between HbA1c levels and the number of visual field deterioration points in the group with a longer duration of diabetes is indeed very interesting. However, could it be that cases with a longer duration of diabetes and poor HbA1c control inherently have worse visual field impairment compared to other cases? In other words, is it possible that the visual field impairment due to diabetes has already reached its peak in these cases? If you wish to assert, as mentioned on line 270, that 'a higher HbA1c was associated with a lower number of deteriorated test points,' it would first be necessary to examine whether there is a significant difference in baseline visual field impairment between the groups stratified by duration of diabetes.

**Reply:** The analysis of risk factors for the early deterioration found a slower deterioration rate with worse HbA1c in cases with long duration. Thus, the deterioration did not seize. We therefore do not think it is possible that the impairment has reached a peak. We have not stratified the baseline data because it renders too few cases for a statistical inference analysis, e.g. only 11/56 cases had diabetes duration > 25 years at baseline.

-------------------

## Comments and replies to Reviewer #2

Title: Ten Years of visual Field Change in Patients with Diabetes: A prospective longitudinal Study-better edited as: Ten years of visual field change in patients with diabetes: A prospective longitudinal study

**Reply:** Capital letters have been changed to lowercase letters as suggested.

-------------------

Abstract
The abstract must fit with Plos one guideline

**Reply:** The abstract has been changed accordingly.

-------------------

Introduction
Included the magnitude of diabetes, and DR globally and in your country as well

**Reply:** A new paragraph to set the scene has been added to the Introduction, Manuscript with Track Changes, line 75 - 83:

“Worldwide 537 million people were estimated to have diabetes in 2021 corresponding to a prevalence rate of 10.5 % [1]. In Sweden the prevalence rate is estimated to reach above 10% in 2050 [2]. Diabetic retinopathy is a common retinal microvascular complication to diabetes with a global prevalence of 22 % [3]. In Sweden DR is present in approximately 1/3 of people with diabetes [4,5]. Controlling blood glucose and blood pressure can postpone DR and local eye treatment with intravitreal injections, laser and surgery can reduce blindness when DR has become sight-threatening. Knowledge how diabetes affects the retina at an early stage could potentially be useful to better monitor DR and develop new treatment strategies aiming to prevent the later sight-threatening stages [6,7].”

And the new references have been added to References:

1. Sun H, Saeedi P, Karuranga S, Pinkepank M, Ogurtsova K, Duncan BB, et al. IDF Diabetes Atlas: Global, regional and country-level diabetes prevalence estimates for 2021 and projections for 2045. Diabetes Res Clin Pract. 2022;183:109119. doi: 10.1016/j.diabres.2021.109119

2. Andersson T, Ahlbom A, Carlsson S. Diabetes Prevalence in Sweden at Present and Projections for Year 2050. PLoS One. 2015;10(11):e0143084. doi:10.1371/journal.pone.0143084

3. Teo ZL, Tham YC, Yu M, Chee ML, Rim TH, Cheung N, et al. Global Prevalence of Diabetic Retinopathy and Projection of Burden through 2045: Systematic Review and Meta-analysis. Ophthalmology. 2021;128(11):1580-1591. doi:10.1016/j.ophtha.2021.04.027

4. Olafsdottir E, Andersson DK, Dedorsson I, Stefánsson E. The prevalence of retinopathy in subjects with and without type 2 diabetes mellitus. Acta Ophthalmol. 2014;92(2):133-7. doi:10.1111/aos.12095

5. Sharif A, Smith DR, Hellgren KJ, Jendle J. Diabetic retinopathy among the elderly with type 2 diabetes: A Nationwide longitudinal registry study. Acta Ophthalmol. 2024;102(6):e883-e892. doi:10.1111/aos.16659

6. Antonetti DA, Klein R, Gardner TW. Diabetic retinopathy. N Engl J Med. 2012;366(13):1227-39. doi:10.1056/NEJMra1005073

7. Stitt AW, Curtis TM, Chen M, Medina RJ, McKay GJ, Jenkins A, et al. The progress in understanding and treatment of diabetic retinopathy. Prog Retin Eye Res. 2016;51:156-86. doi:10.1016/j.preteyeres.2015.08.001

-------------------

The relationship between Visual field function and DR change must be clearly stated.

**Reply:** A change (improvement or deterioration) in visual function and the development of DR can only be explored in longitudinal studies. The relationship has been further clarified in the introduction, Manuscript with Track Changes, line 91 – 101, especially at the end of the paragraph line 96 - 101.

From: “However, longitudinal studies are needed to investigate whether change in visual function correlates to change in DR, and what risk factors are important for such early change in function.”

To: “However, longitudinal studies are compulsory to investigate whether subtle changes deterioration or improvement in visual function, correlate or precede microvascular damage. Such subtle changes are difficult to detect by only arbitrary examine graphic test results e.g. grey scales of raw threshold values.”

-------------------

Method
What was your eligibility criteria?

**Reply:** A clarification has been made in the Methods section, Manuscript with Track Changes, line 127-129:

“Eligibility criteria were diabetic subjects aged 18 to 75 years, who attended the screening program for DR in the county of Värmland, for more details see Hellgren et al. 2013 [19].”

-------------------

The selection criteria is not clear?

**Reply:** The participants were consecutively recruited from the local screening program for DR with eligibility criteria above.

-------------------

How do you reassure that the participants do not have a visual change at baseline?

**Reply:** In this manuscript we consider visual field deterioration or improvement over time, i.e. change from the study start, which is not to the same as visual field change compared to a normal visual field. Visual field change due to diabetes may have occurred before inclusion in the study especially in participants with long diabetes duration.

A clarification has been inserted in the Introduction section, Manuscript with Track Changes, line 96 – 101:

From: “However, longitudinal studies are needed to investigate whether change in visual function correlates to change in DR, and what risk factors are important for such early change in function.”

To: “However, a longitudinal study design is compulsory to investigate whether subtle changes, deterioration or improvement, in visual function correlate or precede retinal microvascular damage. Such subtle changes are difficult to detect by only arbitrary examine graphic test results e.g. grey scales of raw threshold values.”

-------------------

When does the baseline assessment done?

**Reply:** The baseline assessment was done within a few weeks from recruitment, from Oct 11, 2006, until Sept 17, 2009.
 -------------------

DR is the most common microvascular complication in T1DM, but less likely in T2DM? but most of your participants were T2DM, why?

**Reply:** Most patients had type 2 diabetes, since in Sweden type 2 diabetes is far more common than type 1, and patients were consecutively recruited from the screening program for DR which is open for both type 1 and type 2. This recruitment strategy was chosen to provide a representative cohort of patients with diabetes who typically are monitored by retinal microvascular morphology only.

-------------------
Line 123: similar to the that….editing

**Reply:** The sentence has been edited, Manuscript with Track Changes, line 157 - 158:

“… similar to the model commonly used for detection of visual field deterioration caused by glaucoma.”

-------------------

What was the sample size including sample size calculation

**Reply:** In the current study the sample consisted of participants who previously had completed a five-year follow-up, n = 63 (Hellgren et al. in Diabetes, 2014;63:3104–3111 and Acta Ophthalmol, 2013;91:672–677). Of these 63 were 56 included in this continuation. Thus, we did not perform any sample size calculation for analysis/results of the current study. A detailed description is found in the Results section , line 217 - 220. Because this is a continuation of the previous study a sample size calculation was not done.

-------------------

What was the sampling procedure?

**Reply:** A systematic sampling was performed by consecutively recruiting the diabetic subjects from the local screening program.

-------------------

There must be a clear operational definition of Visual field change and DR

**Reply:** Regarding visual field change, the model identifies test points in the visual field which have changed, deteriorated or improved, from baseline at the 5 % significance level.

A clarification has been made in the method section to the sentence, Manuscript with Track Changes, line 162 - 164:

From: “With knowledge of such random test-retest variability, the model identifies change by comparing visual fields with baseline field tests.”

To: “With knowledge of such random test-retest variability the model identifies change, deterioration or improvement, by comparing each follow-up visual field test result with that obtained at baseline, and flag test points which changed significantly at the p < 0.05 level.”

Regarding DR change, a change required two steps change on the ETDRS severity scale except or if retinopathy level changed between level 10, no retinopathy, to level 20, microaneurysms only, A clarification has been made to the sentence, Manuscript with Track Changes, Line 188 - 190.

From:  “Progression of DR change required an increase of at least two steps on the ETDRS severity scale or if retinopathy level changed from level 10, no retinopathy, to level 20, microaneurysms only.”

To: “A DR change required a change of at least two steps on the ETDRS severity scale or if retinopathy level changed from level 10, no retinopathy, to level 20, microaneurysms only, or vice versa.

-------------------

A multivariable model included all potential factors looks incomplete please rewrite it.

**Reply:** The sentence has been clarified, Manuscript with Track Changes, line 214-215.

From: “A multivariable model included all potential factors.”

To: “A multivariable model included the potential factors mentioned above.”

-------------------

Better rewrite the method parts section by section. As study setting, design, population (including and excluding), sample size and sampling procedure, data collection tools and procedures, and method of analysis.

**Reply:** We have included several subheadings to clarify the structure of the Method section. Because this was an extension of a previous study the sample size and sampling procedure was bound to those patients who had completed the previous five-year study. We thank the reviewers for all the suggested changes in the method section.

-------------------

What was the data collection tools and procedures, State clearly in the method section
Who collect the data?

**Reply:** The measurement tools and procedures are described under subheading “Measures”, line 140 - 190.

-------------------

How do you control a visual field change by other causes?

**Reply:** At each visit any changes in ocular and systemic history was noted. If the updated history or the examination indicated visual field change by other causes the patient was examined by an ophthalmologist including a slit lamp examination and ophthalmoscopy. A clarification has been inserted in the Method section, subheading Measures, Manuscript with Track Changes, line 150 – 152:

Ocular and medical history was updated at each visit. If the updated history or examination indicated a visual field change by causes other than diabetes, the patient was examined by an ophthalmologist, including a slit lamp examination and ophthalmoscopy.

-------------------

Why you prefer GEE other than LMM?

**Reply:** Because our outcome was count data and therefore not suitable for a linear model. Generalized estimating equations is a more flexible method which allows to handle count data in Poisson and negative binomial models.

-------------------

Result
Of those eight who did not participate this is not correctly calculated
Line 180….From line….from

**Reply:** Thank you, the text has been corrected, Manuscript with Track Changes, line 218.

From: “Of those eight who did not participate, …”

To: “Of those seven who did not participate, …”

-------------------

Table 1: HbA1c, why do not use %?

**Reply:** Departments of laboratory medicine in Sweden use the International Federation of Clinical Chemistry (IFCC) standardization and delivers the HbA1c result as mmol/mol. The international system of units seems likely appropriate for an international audience. Percent may be ambiguous because they differ between NGSP and Mono-S, whereof the latter were used in Sweden prior to IFCC. Finally, because the journal did not claim values to be reported in %.

-------------------

Diabetes treatment (n) four of them under this category lacks clarity. So try to rewrite it clearly.

**Reply:** The categories have been clarified as Diet only/Non-insulin hypoglycemic agents only/Non-insulin hypoglycemic agents and insulin/Insulin only.

-------------------

Line 190-there is controversies 79% Vs 68%,

**Reply:** The sentence has been clarified, Manuscript with Track Changes, line231 – 233:

From: “Most eyes had no or only mild DR, 44/56 (79%) at baseline and 38/56 (68%) at the last visit, (Fig 1).”

To: “Most eyes had no DR, ETDRS level 10, or microaneurysms only, ETDRS level 20, both at baseline and at the last visit, 44/56 (79%) and 38/56 (68%) respectively, while higher levels of retinopathy were less common, (Fig 1).

-------------------

Line 197, three study eyes not clear

**Reply:** Apologies, a full stop was missing. A full stop has been inserted between the sentences, Manuscript with Track Changes, line 239:

“The regressions occurred most often from ETDRS level 20 to level 10, 27/29 ( 93%). Three study eyes, …”

-------------------

Line 205: needs subtitle as Visual field ……..

**Reply:** A subheading has been inserted as suggested, Manuscript with Track Changes, line 248.

-------------------

207 IRR….describe it as long form with short form then proceed with short form

**Reply:** The manuscript has been changed accordingly. The long form is written at first occurrence in the Method section, subheading Statistics, Manuscript with Track Changes, line 204 – 205, and then as the short form.

-------------------

Line 223-225, Is it COR or AOR? Is it recommended in this study?
Make a consistent description……in DR

**Reply:** The odds ratio was adjusted for time and a clarification has been made in the Method section, subheading Statistics, Manuscript with Track Changes, line 208.

From: “To analyze whether deteriorated test points were associated with DR change, we applied a logistic model.”

To: “To analyze whether deteriorated test points were associated with DR change, we applied a logistic model controlling for the effect of time.”

-------------------

Line 230-232; The number 2.4 % and 2.1% are not correct based on the CIs stated.

**Reply:** The numbers refer to the average IRR, a clarification has been made including CI, Manuscript with Track Changes, line 274 -  276.

From: Thus, an increase of 2.4% deteriorated test points for each mmol/mol higher blood pressure and a decrease of 2.1% deteriorated test points for each mmHg higher blood pressure.

To: Thus, an average increase of 2.4% (0.7 – 4.2) deteriorated test points for each mmol/mol higher HbA1c and an average decrease of 2.1% (0.1 – 4.0) deteriorated test points for each mmHg higher blood pressure.

-------------------

Discussion
Lacks novelty need to be rewrite it
Start with the aim of the study, then proceed to your hypothesis
Use consistent referencing line 281 and 288
Please include the study implications and recommendations,
Conclusion better stated separately with the discussion and stick to your findings

**Reply:** Thank You for this comment which helped us to improve the discussion which has been re-written and restructured. It now highlights the novelty of the study and follows the suggested structure and change.

-------------------

The entire manuscript need to be edited by professional language expert

**Reply:** The manuscript was edited by a professional language expert before submission.
