# Supplementary material for: Ten years of visual field change in people living with diabetes: A prospective longitudinal study
Source: PLoS One. 2025 Mar 25;20(3):e0320285. doi: 10.1371/journal.pone.0320285 (PMC11936255; doi:10.1371/journal.pone.0320285)
Supplement: Attachment — Submitted filename: Response to Reviewers 2nd rev.docx [file pone.0320285.s002.docx]

Thank you for the opportunity to improve the manuscript by additional changes. Please, se our replies below.

Yours sincerely,

Karl-Johan Hellgren, MD, PhD

## Comments and replies

### Journal Requirements:

Please review your reference list to ensure that it is complete and correct. If you have cited papers that have been retracted, please include the rationale for doing so in the manuscript text or remove these references and replace them with relevant current references. Any changes to the reference list should be mentioned in the rebuttal letter that accompanies your revised manuscript. If you need to cite a retracted article, indicate the article’s retracted status in the References list and also include a citation and full reference for the retraction notice.

**Reply**: The reference list has been reviewed. A correction has been made in the Revised Manuscript with Track Changes, Method section, subheading Statistics, Line 204, from [16] to [23]. This correction did not imply any changes to the reference list. Seven references were added in the first revision to include an introductory paragraph in the introduction regarding the magnitude of diabetes and DR globally and in Sweden. Two references were excluded in the first revision, but these are now included again (Simo 2018 Diabetologia and van der Kreke, PLOS One 2020). Thus, the reference list in this second revision is correct with 34 references. No papers have been retracted.

### Comments and replies to Reviewer #1:

1. If the distinction between type 1 and type 2 diabetes did not impact the results, it might not be necessary to separate them strictly. To avoid potential inaccuracies in the manuscript, defining individuals diagnosed with diabetes under the age of 30 as having 'early-onset diabetes' could be a clearer and more precise alternative.

**Reply**: The suggestion has been adopted. Revised Manuscript with Track Changes, Method section, subheading Study design, line 132-133 and Result section, line 218.

-------------------

2. Apologies for missing this in the initial review, but the title of Figure 1 appears to be just the legend, which seems very unusual. Perhaps the title could be something like 'ETDRS levels at the first visit and the final visit of the patients' to make it clearer.

**Reply**: A clarification has been made as suggested. Revised Manuscript with Track Changes, Result section, line 236-237.

Comments and replies to Reviewer #2

Purpose ….better change to Background /introduction based on the journal guideline. see the previous comments

**Reply**: Additional subheadings now clarify the abstract structure. We hope this is sufficient but are not entirely sure because we find abstracts without and with varying subheadings in PLOS One. We have considered the guidelines and the advice from the following sources: <https://journals.plos.org/plosone/s/submission-guidelines#loc-abstract>

and <https://plos.org/resource/how-to-write-a-great-abstract/>

-------------------

Change the diabetes patients as “people living with diabetes”

**Reply**: The term people living with diabetes is a respectful, including and formal term. The entire manuscript has been changed accordingly.

-------------------

The IQR should be stated in range form not the subtraction result, so edit the entire manuscript table

**Reply**: The IQR has been changed from subtraction to the range form.

-------------------

The study design must be expressed in the study design section

**Reply**: A clarification has been added. Revised Manuscript with Track Changes, Method section, subheading Study design, line 127.

-------------------

Include the study implication in the discussion section

**Reply**: Implications have been further clarified. Revised Manuscript with Track Changes, Discussion section, line 348-353.

-------------------

Please use conclusion as separate a title after discussion.

**Reply**: A subheading has been added as suggested.
